# Astrocyte elevated gene-1(AEG-1) induces epithelial-mesenchymal transition in lung cancer through activating Wnt/β-catenin signaling

**DOI:** 10.1186/s12885-015-1124-1

**Published:** 2015-03-08

**Authors:** Weiling He, Shanyang He, Zuo Wang, Hongwei Shen, Wenfeng Fang, Yang Zhang, Wei Qian, Millicent Lin, Jinglun Yuan, Jinyang Wang, Wenhua Huang, Liantang Wang, Zunfu Ke

**Affiliations:** 1 Department of Gastrointestinal Surgery, Guangzhou, 510080 Province Guangdong People's Republic of China; 2 Gynecology, and the First Affiliated Hospital of Sun Yat-sen University, Guangzhou, 510080 Province Guangdong People's Republic of China; 3 Department of Pathology, the First Affiliated Hospital, Sun Yat-Sen University, 58 Zhongshan Road II, Guangzhou, Guangdong 510080 People's Republic of China; 4 Department of Oncology, Sun Yat-sen University CancerCenter, Guangzhou, 510060 Province Guangdong People's Republic of China; 5 College of Engineering, University of Texas, El Paso 500 West University Avenue, El Paso, TX 79968 USA; 6 Department of Molecular and Medical Pharmacology, University of California, Los Angeles, 570 Westwood Plaza, Los Angeles, CA 90095-1770 USA; 7 Department of Anatomy, School of Basic Medical Science, Southern Medical University, Guangzhou, Guangdong 510515 People's Republic of China

**Keywords:** AEG-1, Epithelial-mesenchymal transition, Non-small cell lung cancer, Wnt, β-catenin

## Abstract

**Background:**

Non-small cell lung cancer (NSCLC) is a highly metastatic cancer with limited therapeutic options, so development of novel therapies that target NSCLC is needed. During the early stage of metastasis, the cancer cells undergo an epithelial-mesenchymal transition (EMT), a phase in which Wnt/β-catenin signaling is known to be involved. Simultaneously, AEG-1 has been demonstrated to activate Wnt-mediated signaling in some malignant tumors.

**Methods:**

Human NSCLC cell lines and xenograft of NSCLC cells in nude mice were used to investigate the effects of AEG-1 on EMT. EMT or Wnt/β-catenin pathway-related proteins were characterized by western blot, immunofluorescence and immunohistochemistry.

**Results:**

In the present study, we demonstrated that astrocyte elevated gene-1(AEG-1) ectopic overexpression promoted EMT, which resulted from the down-regulation of E-cadherin and up-regulation of Vimentin in lung cancer cell lines and clinical lung cancer specimens. Using an orthotopic xenograft-mouse model, we also observed that AEG-1 overexpression in human carcinoma cells led to the development of multiple lymph node metastases and elevated mesenchymal markers such as Vimentin, which is a characteristic of cells in EMT. Furthermore, AEG-1 functioned as a critical protein in the regulation of EMT by directly targeting multiple positive regulators of the Wnt/β-catenin signaling cascade, including GSK-3β and CKIδ. Notably, overexpression of AEG-1 in metastatic cancer tissues was closely associated with poor survival of NSCLC patients.

**Conclusions:**

These results reveal the critical role of AEG-1 in EMT and suggest that AEG-1 may be a prognostic biomarker and its targeted inhibition may be utilized as a novel therapy for NSCLC.

## Background

Lung cancer is the most common malignant tumor in the world, and the leading cause of cancer-related death in human beings [1]. Despite the achievements made in diagnosis and treatment in the recent years, the prognosis of lung cancer patients is still poor and their overall 5-year survival rate is 15% [2]. Although the clinical stage at diagnosis is the key prognostic determinant for lung cancer survival [3], considerable variability in reoccurrence and survival is commonly observed in patients with a similar stage. Thus, the initial diagnosis is extremely important because it could reduce the mortality rate for lung cancer patients [4].

The progress of cancer metastasis depends on the unique mechanisms of cancer cells evading from the primary tissue and spreading into surrounding tissues. Molecular reprogramming, as a part of the epithelial–mesenchymal transition (EMT), is considered to be a crucial step in the metastasis process of most carcinomas [5]. During metastatic progression, EMT drives primary epithelial-like tumour cells to acquire invasive potential, such as increased motility and mesenchymal characteristics, triggering dissemination from the tumor and infiltration into the tumor vessel. Then, the EMT-driven cells circulating in the blood flow redifferentiate into primary status via MET during colonization and growth at distant metastatic sites [6,7]. Because of EMT’s role in the metastatic process, controlling EMT progress and progression in tumors is now thought to be a promising strategy to inhibit metastasis and to prolong cancer patients’ survival.

Astrocyte-elevated gene-1 (AEG-1), also known as LYRIC (lysine-rich CEACAM1) or metadherin, is originally induced in primary human fetal astrocytes [8]. Recently, numerous reports demonstrated that AEG-1 might play a pivotal role in the pathogenesis, progression, invasion, metastasis and overall patient survival in diverse human cancers [9-12]. This evidence indicates that the upregulation of AEG-1 contributes to malignant progression [13]. Furthermore, AEG-1 overexpression can facilitate migration and invasion of human glioma cells [14], as well as activate Wnt/β-catenin signaling via ERK42/44 activation [11]. Although AEG-1 is an oncogene that has been implicated in pathways critical to lung cancer carcinogenesis [15], AEG-1 was also found to control the expression of E-cadherin and Vimentin [16]. The above findings suggest that AEG-1 may mediate the metastasis of lung carcinoma through the regulation of EMT.

In this study, we concentrated on elucidating the role of AEG-1 in EMT of NSCLC. We demonstrated that upregulation of AEG-1 was significantly associated with lymph node metastasis and EMT status of NSCLC. We further investigated that AEG-1 could activate Wnt/β-catenin signaling by inducing GSK-3β (glycogensynthasekinase 3β) phosphorylation via CKIδ (casein kinase Iδ), consequently enhancing EMT status.

## Methods

### Cell culture and tissue specimen selection

Lung cancer cell lines, including NCI-H226, NCI-H460, L-78, A549 and Slu-01, were maintained in Dulbecco’s modified Eagle’s medium (DMEM; Invitrogen, USA) supplemented with 10% fetal bovine serum (HyClone, Logan, UT). AEG-1 overexpression plasmid pcDNA3.1-AEG-1, β-catenin overexpression plasmid pcDNA3.1-β-catenin, AEG-1 siRNA and CKIδ siRNA (RiboBio, China) were transiently transfected using Lipofectamine 2000 (Invitrogen, USA).

A total of 210 cases from 2000 to 2005 coded as “lung cancer” were collected consecutively from the pathology archives of the Affiliated First Hospital, Sun Yat-sen University. The medical ethics committee of Sun Yat-sen University approved the present retrieval of cancer specimens and the connection with clinical data from our institute.

### Migration assay

Invasive ability was measured by using 24-well BioCoat cell culture inserts (Costar, New York, NY, USA) with an 8-μm-porosity polyethylene terephthalate membrane coated with Matrigel Basement Membrane Matrix (Cultrex, MD, USA). At the end of the assay, cells that did not migrate or invade through the pores were removed with a cotton swab. The invasion ability was determined by counting the cells that migrated to the lower side of the filter.

### Western blot and immunofluorescence

Western blot was carried out as described earlier [17]. Blotted membranes were incubated with the antibodies for AEG-1(Invitrogen, USA), Twist 1, E-cadherin, Vimennt, β-catenin, p-GSK-3β(Ser-9), GSK-3β, CKIδ and GAPDH (Abcam, Cambridge, UK) in 5% milk/TBST (tris-buffered saline Tween-20). For immunofluorescence microscopy, cells grown on chamber slides were probed with AEG-1 E-cadherin, Vimennt and β-catenin. The fluorescein isothiocyanate (FITC)-conjugated or rhoda-mine-conjugated anti-IgG was purchased from Molecular Probes. Cells were visualized in an Olympus BX51 fluorescence microscope (Olympus, Tokyo, Japan).

### Total RNA extraction and real-time RT-PCR

Total RNA was extracted using the RNAeasy kit (Qiagen, USA). The amplification was carried out in a total volume of 20 μL containing LightCycler FastStart DNA Master SYBR green I (Roche, USA). Ct value (initial amplification cycle) of each standard dilution was plotted against standard cDNA copy numbers. On the basis of the standard curves for each gene, the sample cDNA copy number was calculated according to the sample Ct value. Standard curves and PCR results were analyzed using ABI7000 software (Applied Biosystems, Foster City, CA, USA). Primers were β-catenin: (sense) 5′GTTTCGTTTCCGCTGTTA 3′, (antisense)5′ TTTCTCCCTCTTGCCATC 3′ and AEG-1: (sense) 5′CGAGAAGCCCAAACCAAATG 3′, (antisense) 5′TGGTGGCTGCTTTGCTGTT 3′. β-actin (primers: sense 5′ GCATGGGTCAGAAGGATTCCT 3′, antisense 5′ TCGTCCCAGTTGGTGACGAT 3′) was used as an internal control.

### Immunoprecipitation

For immunoprecipitation, all of the procedures were done at 4°C. Transfected Slu-01 cells were washed twice with cold PBS and rinsed in 1.5 ml of cold lysis buffer for 20 min on ice. After preclearing, 1 mg of total protein was incubated with antibody, AEG-1,GSK-3β, or CKIδ. An equal concentration of sheep (Upstate Cell Signalling Solutions), mouse, or rabbit (Vector laboratories) immunoglobulin was used as controls. The immunocomplexes were subjected to Western blot analysis according to the manufacturer’s protocol.

### Luciferase reporter gene assay

For the reporter gene assay, cells seeded in 24-well plates were transfected with the firefly luciferase reporter gene construct (TOP or FOP; 200 ng), and 1 ng of pRL-SV40 Renilla luciferase (as an internal control). Cell extracts were prepared 24 hours after transfection, and luciferase activity was measured using the Dual-Luciferase Reporter Assay System (Promega, USA).

### Analysis of the Wnt signaling pathway

Wnt-3a-conditioned medium (Wnt-3a-CM) was produced from L cells transfected with pGKWnt-3a. The medium was centrifuged at 1,000 g for 15 min and filtered through a nitrocellulose membrane. Then, cells were treated with Wnt-3a CM for 24 hours, and Wnt signaling was monitored by various assays, including Western blotting and luciferase reporter gene assays.

### Immunohistochemical staining and evaluation

Sections (4 μm) of formalin-fixed, paraffin-embedded tissues were made using a rotary microtome (Leica, Wetzlar, Germany) and labeled with anti-AEG-1 (Abcam, Cambridge, UK), anti-E-cadherin (Abcam, Cambridge, UK) and anti-Vimentin (Abcam, Cambridge, UK) primary antibodies. We used the known positive slice in the SP kit (Maxim-Bio, Fuzhou, China) as a positive control. The number of immunopositive cells was semiquantitatively estimated. The staining index was calculated using Aperio ImageScope software (Aperio Technologies).

### In vivo orthotopic xenograft studies in athymic nude mice

Male nude mice (about 8 weeks of age) were anesthetized with sodium pentobarbital (50 mg/kg) in a sterile environment. A small skin incision to the right chest wall was made approximately 5 mm to the tail side of the scapula. Then, Slu-01 (5 × 106) or Slu-01/AEG-1-expressing cells (5 × 106; Slu-01 cells stably transfected with the human AEG-1 complementary DNA) were implanted into the right lung of individual nude mice using one-milliliter syringes with hypodermic needles. The skin incision was sutured using metallic clips, which were removed on day 16 after the operation. Different time after inoculation, the mice were killed, tumors were weighed and measured, and tumor tissues were fixed in 10% neutral buffered formalin for the immunohistochemical study. For H&E staining, deparaffinized tissue sections were stained with Mayer hematoxylin and eosin solution. Tumor growth and local metastasis were monitored by an IVIS Imaging System (Xenogen). Images and bioluminescent signals were analyzed using Living Image and Xenogen software. All experimental projects were approved by the medical ethics committee of Sun Yat-sen University.

### Statistical analysis

All above experiments were performed at least three times. Statistical analysis was carried out using software SPSS (version 16.0; SPSS, Chicago, IL, USA). Unpaired two-tailed Student’s *t*-test was used to determine the statistical relevance between groups. Survival curves were plotted using the Kaplan-Meier method and compared with the log-rank test. ROC curve analysis was conducted to determine the cutoff point of high or low AEG-1 level and EMT status. Values of P < 0.05 were considered statistically significant.

## Results

### AEG-1 is closely correlated with EMT status in vitro

**Figure 1:**
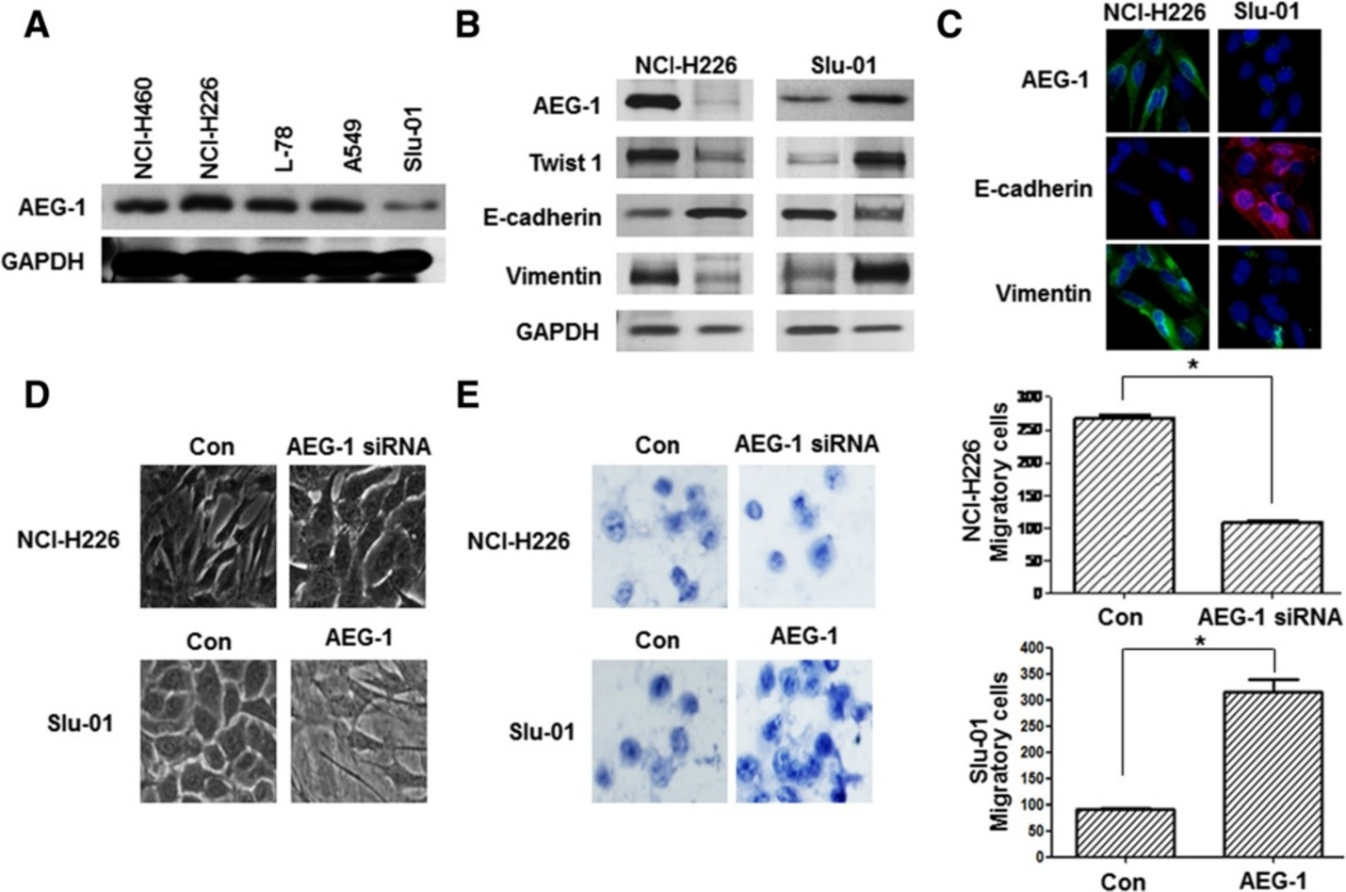
**Lung cancer cell lines showed different AEG-1 expression characteristics and AEG-1 promoted the EMT process. (A)** AEG-1 protein expression levels in lung cancer cell lines. NCI-H226 cells expressed a high level of AEG-1; NCl-H460, L-78, A549 and Slu-1 cells expressed a low level of AEG-1. **(B)** The expression spectrum of mesenchymal and epithelial markers in AEG-1-knockdown cells and pcDNA3.1-AEG-1-transfected cells was analyzed by using the Western blotting method. GAPDH served as a control. **(C)** Immunofluorescence staining of AEG-1, E-cadherin and Vimentin in NCI-H226 and Slu-01 cell lines (magnification × 100). **(D)** Knockdown of AEG-1 reversed EMT in vitro. Morphology of NCl-H226 cells transfected with AEG-1 siRNA was observed through phase-contrast microscopy (magnification × 100). Up-regulation of AEG-1 initiated EMT in vitro. Slu-01 cells were transfected with pcDNA3.1-AEG-1 and the morphology was observed through phase-contrast microscopy (magnification × 100). **(E)** The effect of AEG-1 expression changes on invasion ability. NCI-H226 cells were transfected with AEG-1 siRNA, and Slu-01 cells were transfected with pcDNA3.1-AEG-1. The data represent the mean ± SD of three independent experiments (asterisk; *p* < 0.01).

To investigate the role of AEG-1 expression in lung cancer, we comparatively analyzed AEG-1 protein profiles in lung cancer cell lines with different metastatic ability. As shown in Figure 1A, Western blot analysis revealed that AEG-1 protein was highly expressed in NCI-H226 cells (from lung squamous cell carcinoma with high metastatic ability), whereas Slu-01 cells (from lung adenocarcinoma with low metastatic ability) had undetectable AEG-1 protein expression. In cell lines (with middle metastatic ability) such as NCI-H460, L-78 and A549, the expression levels of AEG-1 protein were significantly lower than that of NCI-H226 cells, but higher than that of Slu-01 cells. We also showed that NCI-H226 cells expressed high levels of Twist1, Vimentin and E-cadherin, but low level of E-cadherin, while Slu-01 cells displayed the opposite expression pattern (Figure 1B and C). These results indicated that AEG-1 might be associated with the metastasis process of lung cancer.

In addition, of particular note was the fact that AEG-1 could regulate EMT. EMT may aberrantly take place in epithelial neoplasms, leading to the loss of cell polarity, cell-to-cell contact and enhanced cell motility. At the early metastatic stage of tumors, EMT is characterized by the loss of E-cadherin expression, an increase in motility, invasive potential and mesenchymal characteristics such as Vimentin. These phenotypic changes were also observed in NCI-H226 cells transfected with AEG-1 siRNA, which displayed a clear morphological transition from spindle-like fibroblastic (vector control) to cobblestone-like cells with well-organized cell contact and polarity (Figure 1D). The transfection of AEG-1 siRNA resulted in an increase of E-cadherin and a decrease of Vimentin expression in NCI-H226 cells (Figure 1B). On the contrary, AEG-1 overexpression in Slu-01 cells led to a spindle- or star-like morphology in the culture media, as well as a decrease of E-cadherin and an increase of Vimentin expression (Figure 1B and D). These results strongly suggest that AEG-1 may promote a transition from epithelial to mesenchymal phenotype. Matrigel-coated transwell assay also showed that AEG-1 overexpression could significantly enhance cell motility in vitro (Figure 1E).

### AEG-1 promotes β-catenin nuclear translocation and Wnt/β-catenin signaling mediates AEG-1–induced EMT

**Figure 2:**
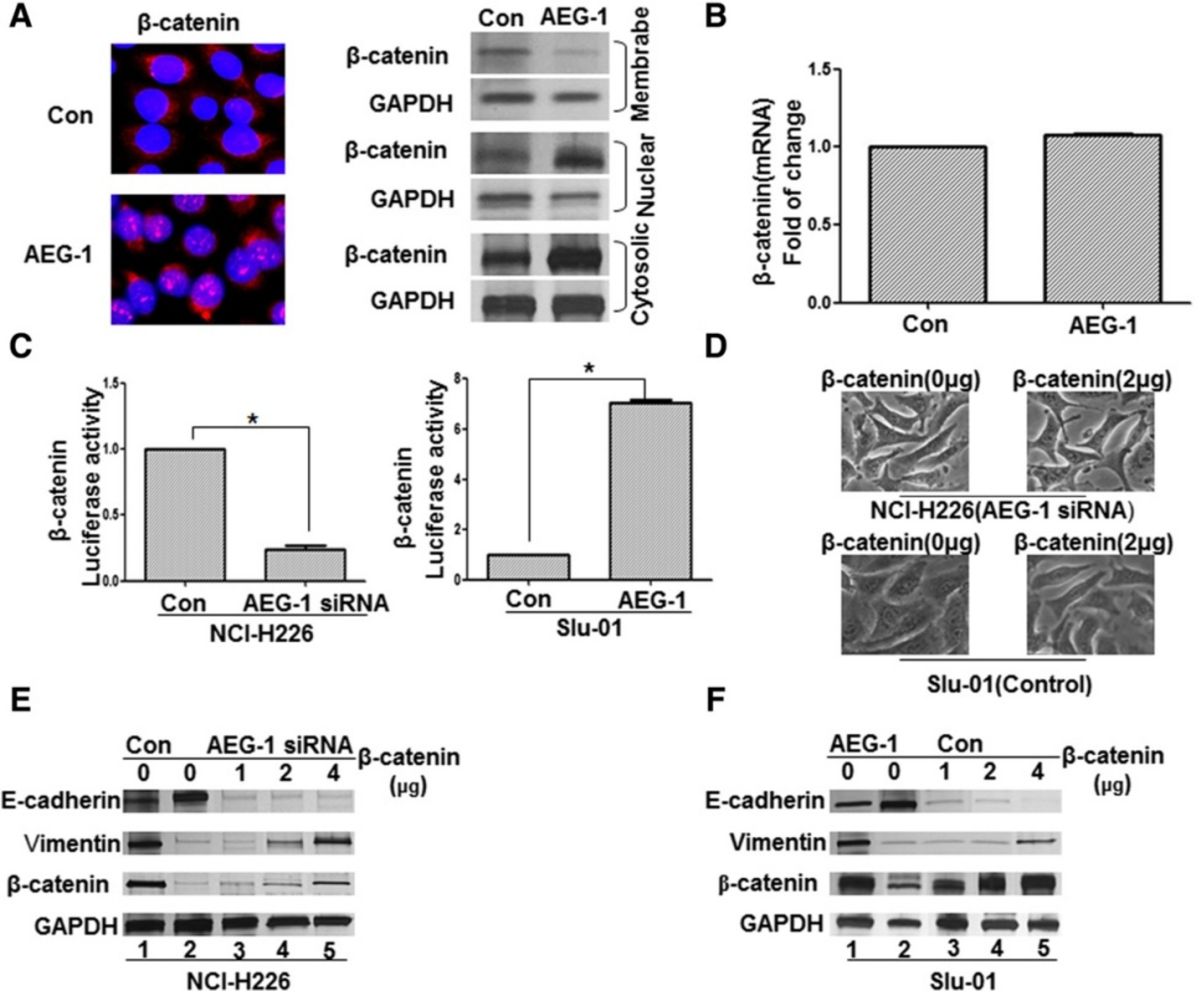
**AEG-1 activated β-catenin, which could reverse AEG-1-siRNA-mediated MET. (A)** AEG-1 promoted β-catenin nuclear translocation. Slu-01 cells were transfected with pcDNA3.1-AEG-1. The subcellular localization of β-catenin was visualized through immunofluorescence (magnification × 400) and Western blotting. **(B)** Total β-catenin mRNA was detected by RT-PCR. **(C)** AEG-1 increased β-catenin/TCF transcriptional activity. NCI-H226 cells treated with AEG-1-siRNA, and Slu-01 cells treated with pcDNA3.1-AEG-1 were transfected with TCF-responsive promoter reporter (TOP-flash) or nonresponsive control reporter (FOP-flash); then, luciferase activity was measured as the ratio of TOP/FOP. Relative luciferase activity is presented as the mean ± SD. (error bars) from each sample after normalizing to the control. The asterisk indicates statistical significance (*p* < 0.01). **(D)** The morphology characteristics of NCI-H226 and Slu-01 cells were observed through phase-contrast microscopy (magnification × 200). **(E)** and **(F)** β-catenin overexpression reverses AEG-1-siRNA-mediated MET. An increasing amount of β-catenin was transfected in NCI-H226 **(E)** and Slu-01 **(F)** cells for 24 hours. Total cell lysates were probed with antibodies against E-cadherin, Vimentin, and β-catenin.

Based on the critical role of the Wnt/β-catenin pathway in metastasis, we then explored whether AEG-1 activates Wnt/β-catenin signaling and if the Wnt/β-catenin pathway mediates AEG-1-induced EMT. In the canonical Wnt/β-catenin pathway, the hallmark of Wnt signaling activation is β-catenin’s nuclear translocation, where it forms a complex with a specific T-cell factor/lymphoid enhancer factor (Tcf/Lef) [18]. After up-regulating AEG-1 expression in Slu-01 cells with pcDNA3.1-AEG-1, we observed a substantial accumulation of β-catenin in the nucleus, suggesting that AEG-1 might contribute to the activation of Wnt signaling (Figure 2A). However, the total β-catenin mRNA level did not change significantly after AEG-1 overexpression in Slu-01 cells (Figure 2B). As expected, luciferase assays also demonstrated that AEG-1 overexpression noticeably increased the transcriptional activity of β-catenin/TCF in Slu-01 cells, as determined by the β-catenin reporter system (TOP/FOP) (Figure 2C). In contrast, transfection of AEG-1 siRNA could reduce the β-catenin/TCF transcriptional activity in NCl-H226 cells (Figure 2C).

If we treated NCl-H226 cells transfected with AEG-1 siRNA with pcDNA3.1-β-catenin, it could restore the EMT status of NCl-H226 cells, as determined by EMT-related marker expression and morphology (Figure 2D and E). Furthermore, elevated expression of β-catenin protein levels in NCI-H226/AEG-1-siRNA cells induced EMT in a dose-dependent manner (Figure 2E and F). However, the opposite phenomenon appeared in the Slu-01/AEG-1 cells with corresponding morphology and EMT-related marker changes (Figure 2D and F).

### AEG-1 interacts with Gsk-3β and CKIδto activate Wnt/β-catenin

**Figure 3:**
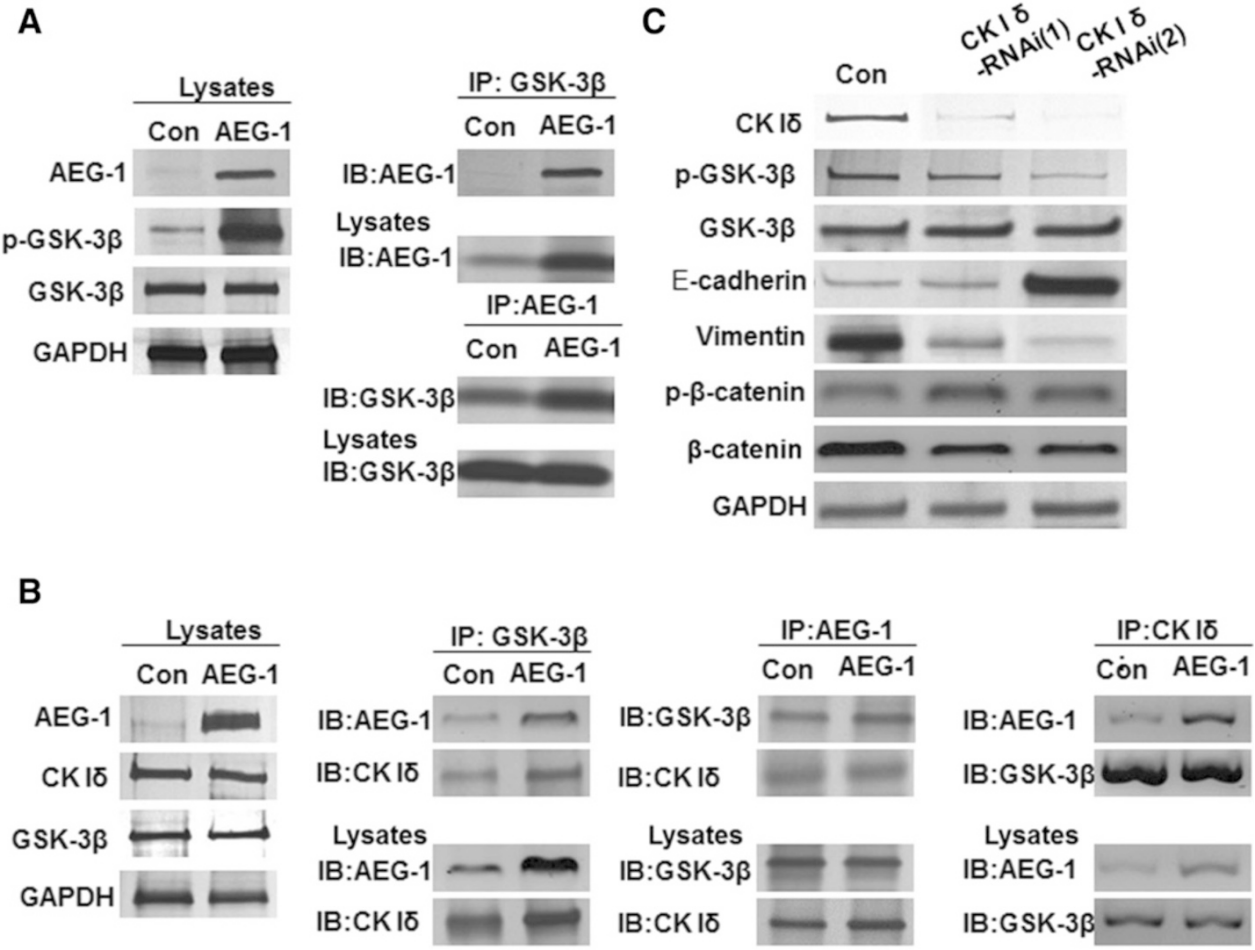
**AEG-1 was associated with CKIδand modulated the GSK-3β/β-catenin signaling pathway. (A)** Slu-01 cells stably overexpressing AEG-1 were established. GSK-3β was immunoprecipitated from cell lysates, and its expression was confirmed by immunoblotting with the indicated antibodies. **(B)** CKIδ was critical for AEG-1-mediated regulation of GSK-3β/β-catenin signaling and EMT. AEG-1 complexes were associated with both GSK-3β and CKIδ. **(C)** CKIδplayed a role in AEG-mediated regulation of EMT and Ser-9 phosphorylation of GSK-3β. Slu-01 cells transfected with pcDNA3.1-AEG-1 were co-transfected with CKIδ-specific siRNA. Cell lysates were then subjected to Western blotting.

We then investigated the molecular mechanism by which AEG-1 activates Wnt/β-catenin signaling. In the absence of Wnt signaling, cytoplasmic β-catenin undergoes sequential phosphorylation, first at Ser^45^(β-cat^45^) by casein kinase I (CKI) and then at Ser^33,37^/Thr^41^ by glycogen synthase kinase (GSK)-3β, leading to targeted ubiquitination through E3 ubiquitin ligase. In Slu-01 cells transfected with pcDNA3.1-AEG-1, immunoprecipitation experiments and Western blot analysis revealed that AEG-1 appeared to directly associate with GSK-3β and promote its phosphorylation at Ser^9^ (Figure 3A). In addition, co-immunoprecipitation results showed that AEG-1 could form a complex with both GSK-3β and CKIδ (Figure 3B). Moreover, after Slu-01/AEG-1 cells were treated with CKIδ-siRNA, CKIδ-siRNA treatment abolished AEG-1-mediated phosphorylation of GSK-3β at Ser^9^ and EMT (Figure 3C).

### AEG-1 promotes Wnt/β-catenin-mediated EMT through inactivating GSK-3β

**Figure 4:**
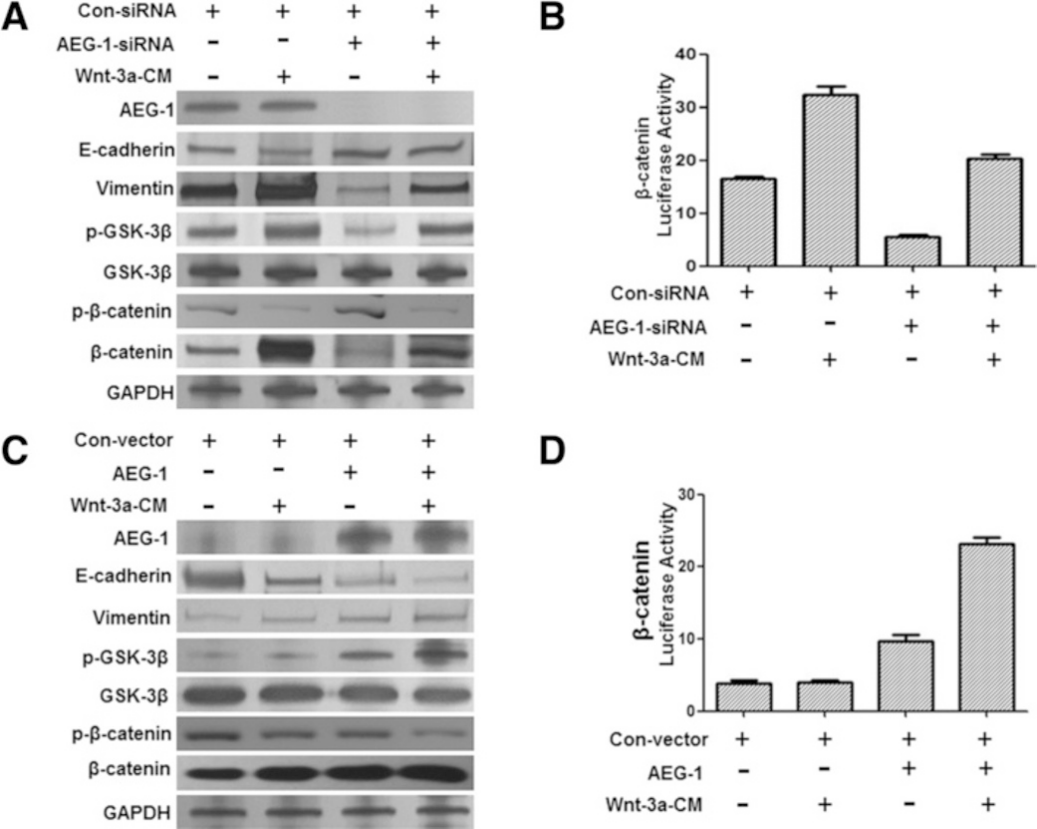
**AEG-1 promoted Wnt-mediated EMT.** Knockdown of AEG-1 activated GSK-3β, and inhibited β-catenin activity and EMT in NCI-H226 cells. Cells were co-transfected with AEG-1-siRNA and TOP or FOP. Then, cells were treated with Wnt-CM. **(A)** The expression of the indicated proteins was analyzed by Western blot in NCI-H226 (control siRNA) and AEG-1-siRNA with or without Wnt-3a-CM, respectively. **(B)** Relative luciferase expression of β-catenin was measured as described above. **(C)** and **(D)** In contrast, restoring AEG-1 expression in Slu-01 cells (AEG-1-negative) promoted Wnt-induced EMT.

Wnt/β-catenin signaling has been demonstrated to participate in the EMT process during embryonic development and cancer progression; however, the involvement of AEG-1 in Wnt/β-catenin-mediated EMT has not been completely defined. To address this question, we tested whether manipulating AEG-1 levels in various cell lines would be able to convert the mesenchymal phenotypes. Whereas Wnt-3a only slightly induced EMT in NCI-H226, AEG-1 depletion notably elicited a change in NCI-H226 cells from the mesenchymal phenotype to an epithelial phenotype as manifested by increased expression of the epithelial marker E-cadherin concomitant with a downregulation of the mesenchymal marker Vimentin (Figure 4A). Similarly, the knockdown of AEG-1 also resulted in the decrease of the p-GSK-3β level and reduced β-catenin/TCF transcriptional activity (Figure 4B).

In contrast, restoring AEG-1 expression in Slu-01 cells (AEG-1-negative cell) reinforced Wnt/β-catenin-induced EMT andled to the increase of the p-GSK-3β level and β-catenin/TCF transcriptional activity (Figure 4C and D), strongly suggesting that AEG-1 is a promoter of Wnt/β-catenin-mediated EMT.

### AEG-1 increases distant metastasis in vivo by the regulation of EMT

**Figure 5:**
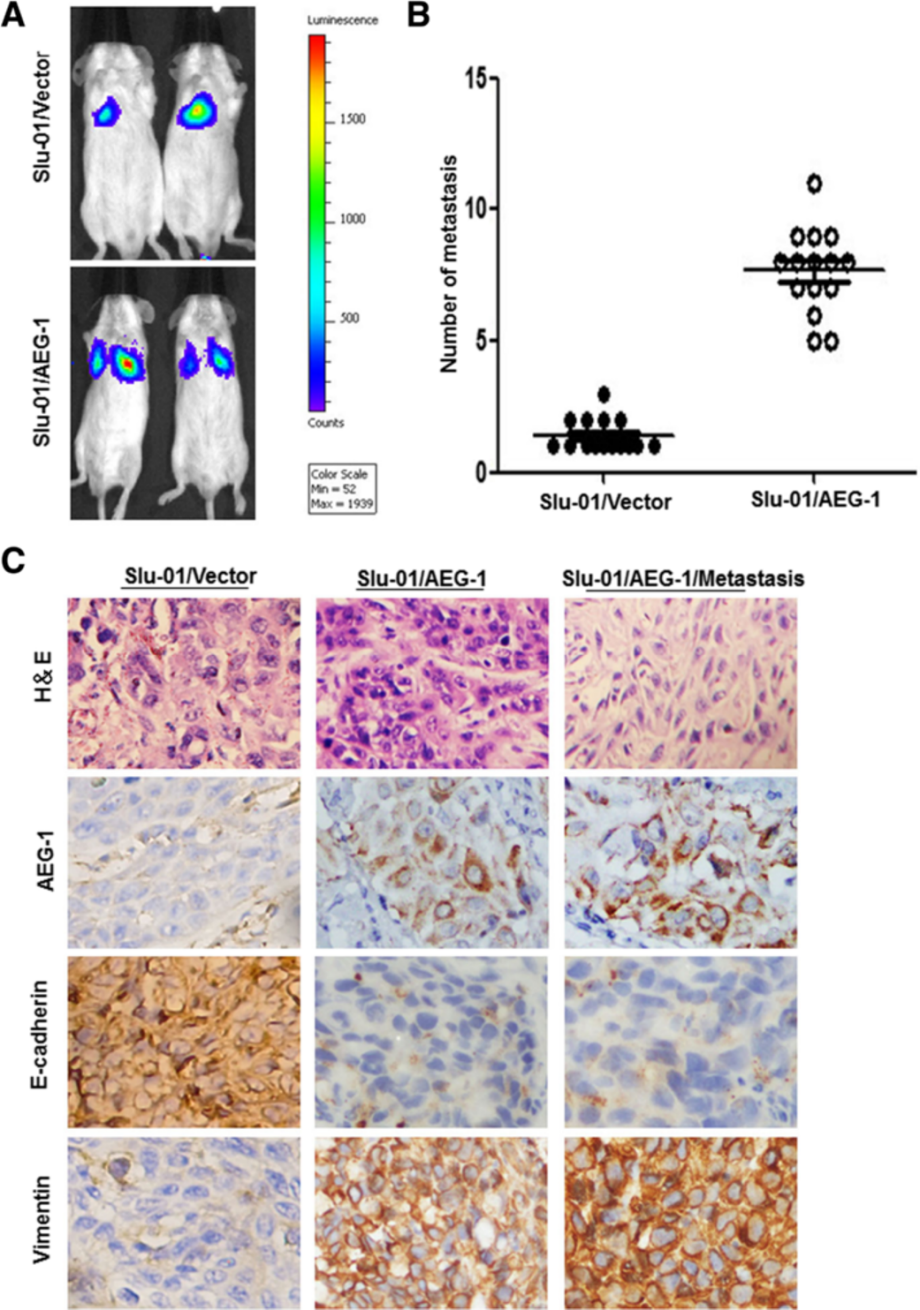
**AEG-1 promoted tumor metastasis in vivo. (A)** Representative BLI images of mice bearing Slu-01/AEG-1-expressing tumors with metastatic lesions. Mice (n = 15) were imaged six weeks later to determine local tumor growth and metastasis. **(B)** Number of metastatic nodules or distant metastasis in individual dead mouse bearing con or Slu-01/AEG-1-expressing tumors. **(C)** AEG-1 overexpression in Slu-01 cells promoted EMT in athymic nude mice in vivo. H&E staining showed primary tumors without detectable metastasis in control mice and the lymph node metastases in mice bearing Slu-01/AEG-1-expressing tumors two weeks after injection (magnification, ×200). IHC showed that up-regulation of AEG-1 resulted in an increased in the expression of Vimentin and weak E-cadherin staining (magnification × 200).

Because Slu-01 cells are of low metastatic potential and decreased AEG-1 expression, and show EMT inhibition status (Figure 1), we then observed the prometastatic trait of AEG-1 up-regulation in Slu-01 cells versus its corresponding vector control cells using an orthotopic mouse model. Stable luciferase activity ensured that every group had an equal level of AEG-1 expression before the injection of Slu-01/AEG-1 cells. Bioluminescent imaging (BLI) was utilized to monitor tumor growth and the onset of metastases dynamically. Strikingly, mice injected with Slu-01/AEG-1 cells displayed multiple distant metastatic lesions at various sites, whereas less metastasis lesions were found in mice injected with control Slu-01 cells (Figure 5A). Our data also showed that Slu-01/AEG-1 xenotransplants approximately generated a 4-fold increase in the number of distant metastases than that of vector control cells (Figure 5B), which was verified by H&E staining (Figure 5C). To further validate the fact that AEG-1 enhanced metastasis in vivo by regulating EMT status, immunohistochemistry(IHC) was applied to detect the expression characteristics of EMT-related molecular markers. Immunohistochemistry (IHC) revealed that the majority of tumor cells in Slu-01/AEG-1 xenotransplants strongly expressed Vimentin, but exhibited weak staining of E-cadherin (Figure 5C).

### AEG-1 promotes metastasis in lung cancer patients

**Figure 6:**
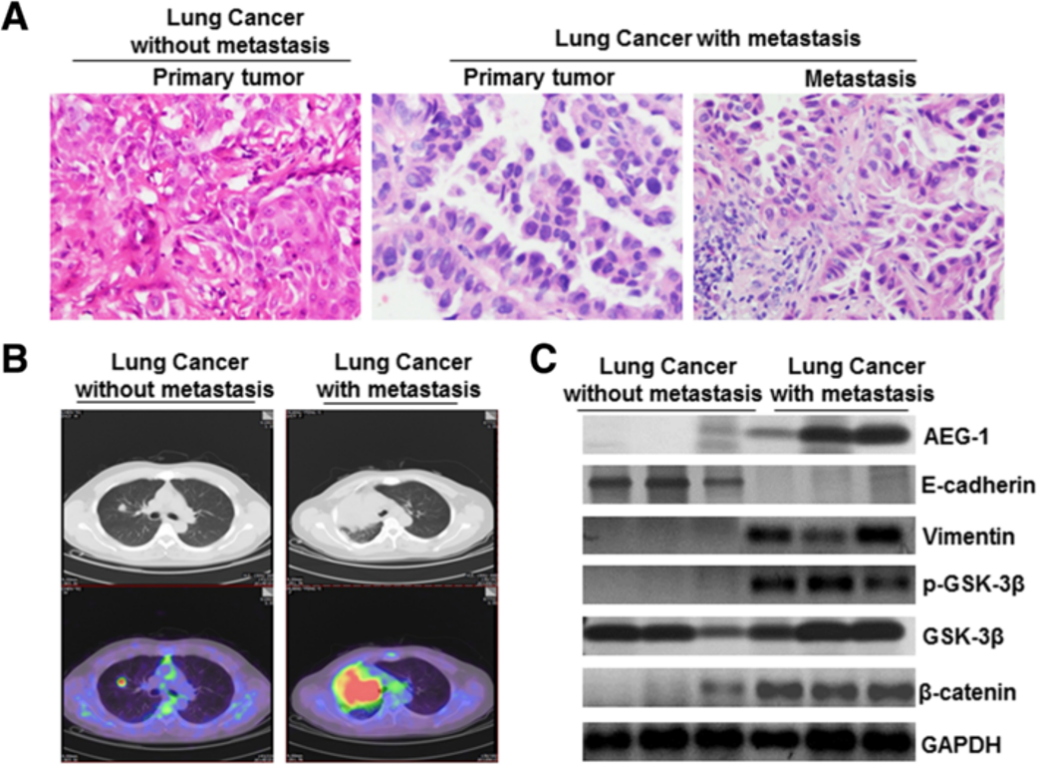
**AEG-1 expression levels were closely correlated with risk of lymph node metastasis in primary cancer lung cancer. (A)** H&E staining was used to identify lung cancer patients with or without lymph node metastasis (magnification × 200). **(B)** AEG-1 regulates EMT in the different stages of lung cancer, including lung cancer patients with or without metastasis, as determined by positron emission tomography/computed tomography. **(C)** AEG-1 over-expression closely correlates with changes in the EMT marker in clinical specimens from lung cancer patients. Expression levels of AEG-1, E-cadherin, Vimentin, p-GSK-3β (Ser-9), GSK-3β and β-catenin in normal (n = 3) and lung cancer (n = 3) tissues were determined by Western blotting. Densitometry was used to determine relative protein levels, and all proteins were normalized to the levels of GAPDH.

To further understand the clinical relevance of the above findings, we examined the relationship between AEG-1 expression and EMT markers in lung cancer patients. Patients from different clinical stages were first divided into two groups according to H&E staining (Figure 6A) and positron emission tomography/computed tomography (PET/CT) (Figure 6B): the primary site of cancer with metastasis and the primary site of non-metastasizing cancer, respectively. Based on the TNM (Tumor node metastasis) staging system, we selected six patients from stage I and stage IV. As shown in Figure 6C, the expression levels of AEG-1 were significantly elevated in patients with distant metastasis, compared to that in primary tumors without detectable distant metastasis. Furthermore, up-regulation of AEG-1, Vimentin, p-GSK-β, and β-catenin levels, as well as suppression of E-cadherin, were clearly observed in tissues from patients with distant metastasis (Figure 6C). In all six examined samples, there was a significantly positive correlation between the levels of AEG-1 and Vimentin and an inverse correlation between the levels of AEG-1 and E-cadherin. These data indicate that AEG-1 plays a pivotal role in lung cancer EMT and metastasis in vivo, which is consistent with our in vitro data from various cancer cell lines.

### Prognostic value of AEG-1 and EMT status in lung cancer patients

**Figure 7:**
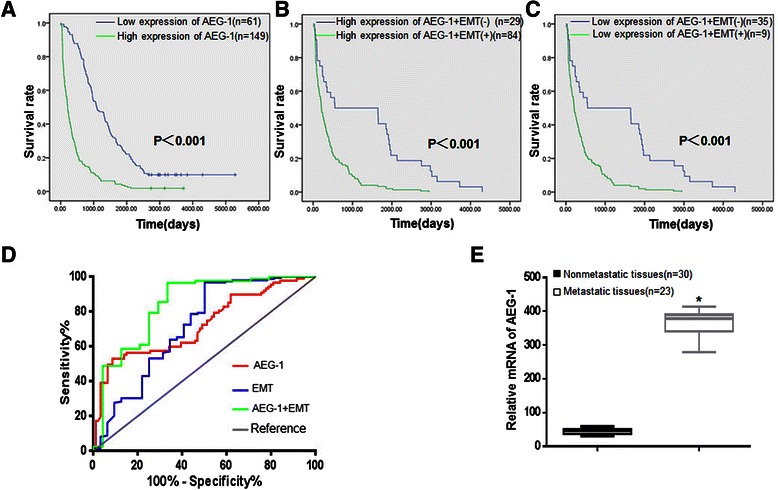
**Kaplan-Meier survival curves according to AEG-1 and EMT status, and corresponding Receiver Operating Characteristic analysis. (A)** The high AEG-1 group correlated with poor survival of lung cancer patients. **(B)** In the AEG-1-positive group, EMT(+) status showed a poor survival trend. Patients with E-cadherin (−) and Vimentin(+) were evaluated as EMT(+); Conversely, patients with E-cadherin (+) and Vimentin(−) were evaluated as EMT(−). **(C)** In the AEG-1-negative group, EMT(+) status also showed a poor survival trend. **(D)** The combined AEG-1 and EMT status had the largest area under the curve compared with AEG-1 level and EMT status, respectively. **(E)** AEG-1 mRNA was determined by real-time RT-PCR in tumors with metastasis (n = 30) and those without metastasis (n = 23) and levels of AEG-1 mRNA were expressed as (AEG-1/β-actin mRNA ratio). *P < 0.05 versus nonmetastatic tissues.

To explore the prognostic value of AEG-1 in patients, we used the Kaplan-Meier method to evaluate the relationship between the survival curve and AEG-1 expression, as well as EMT status. Survival analysis data indicated a significantly inverse correlation between AEG-1 protein expression level and the overall survival time (p < 0.001), clearly disclosing that higher levels of AEG-1 expression were associated with shorter survival time. As shown in Figure 7A, the cumulative 5-year survival rate was 37.8% (95% CI: 25.8%–49.8%) in the AEG-1 low expression group, whereas it was only 5.3% (95% CI: 4.2%–6.4%) in the AEG-1 high expression group.

In addition, when we combined the expression status of AEG-1 and EMT, the difference of overall survival rate between AEG-1(+)/EMT(−) and AEG-1(+)/EMT(+) was significant (p < 0.001, Figure 7B), and it was similar to the result between AEG-1(−)/EMT(−) and AEG-1(−)/EMT(+) (p < 0.001, Figure 7C). Simultaneously, AEG-1 level and EMT status, and their combined status were further analyzed by the receiver operating characteristic (ROC) method to assess their predictive value for death. As shown in Figure 7D, the combined status of AEG-1 and EMT predicted death with better performance (p < 0.001). The area under the curve for both AEG-1 and EMT status was 0.8394 (95% CI: 0.7361–0.9428), which was larger than that of AEG-1 or EMT status, with areas under the curve of 0.7248 (95% CI: 0.6504–0.7992) and 0.7145 (95% CI: 0.6002–0.8287), respectively (both p < 0.001) (Figure 7D).

To further illustrate the clinical significance of the above findings in human lung cancer, AEG-1 mRNA expression was examined in 53 lung cancer tissue specimens. Patients were first divided into two groups: those with distant metastasis and those without metastasis during the follow-up period, respectively. As shown in Figure 7E, AEG-1 mRNA expression level was significantly up-regulated in 23 patients with distant metastasis, compared to that of 30 patients without detectable distant metastasis. The above results suggest a strong correlation between AEG-1 and distant metastasis.

## Discussion

Cancer metastasis is a complex, multistep process involving the escape of neoplastic cells from a primary tumor (local invasion), the intravasation into the systemic circulation, the establishment of micrometastases, and ultimately, the outgrowth of macroscopic secondary tumors [19]. Metastasis is the leading cause of cancer-related deaths worldwide, particularly in NSCLC. Thus, there is an urgent need for the identification of metastatic factors and understanding of the molecular mechanisms underlying NSCLC. AEG-1 is a novel oncoprotein essential for malignant progression in various types of human cancers [9,13,20-23]. Brown et al. pointed out that AEG-1 expression in HEK293T cells enhanced lung localization of the cells. In addition, the knockdown of AEG-1 or anti-AEG-1 antibody inhibited lung metastasis of 4 T1 cells [24]. However, the role of AEG-1 in mediating lung cancer metastasis remains unknown. In our present study, Western blot analysis showed that AEG-1 levels were strikingly up-regulated in the pleura-metastatic derivatives of NCl-H226 lung cancer cell lines. Moreover, AEG-1 evidently induced nonmetastatic Slu-01 cells to invade and metastasize in vitro and promoted a dramatic increase in the incidence of lymph node metastases in vivo. In addition, AEG-1 is significantly correlated with clinical stage, including stages of lymph node spread (an early stage of metastasis) and distant metastasis in breast cancer [21]. In hepatocellular carcinoma cells, expression of AEG-1 gradually increases from stage I to IV [9]. Our analysis also demonstrated that AEG-1 overexpression was closely correlated with metastatic recurrence in lung cancer patients. Thus, the above evidence provides new insight on the function of AEG-1 as a clinically relevant promoter of tumor metastasis.

EMT, a process by which epithelial cells acquire characteristics of mesenchymal cells, is largely thought to play an important role in invasion and metastasis [25]. During EMT, epithelial cells lose their cell polarity and molecular characteristics, but gain migratory and invasive properties [26]. For example, cells undergoing EMT typically show both an increase in protein abundances of Vimentin and a decrease in E-cadherin [27]. These particular phenotypic changes were also observed in Slu-01 cells, which exhibited an obvious morphological transition from a rounded or cobblestone-shaped, epithelial-like morphology to spindle-shaped fibroblast with the loss of its cell polarity, cell–cell adhesive interactions and junctions when transfected with pcDNA3.1-AEG-1. Moreover, AEG-1 could up-regulate Vimentin and down-regulate E-cadherin expression levels in Slu-01 cells. In contrast, when endogenous AEG-1 expression was knocked down in NCl-H226 cells, EMT was clearly conversed. Furthermore, AEG-1 can enhance the Twist 1 expression, which is a potential EMT regulator. The association between EMT and cancer progression has been revealed in several types of cancer [28,29]. More importantly, a conversion from E-cadherin to N-cadherin showed strong and significant associations with prostate cancer progression [30]. However, less research has been done on the role of EMT in lung cancer. We further assessed the relationship between AEG-1 expression and EMT-related markers in lung cancer patients. The suppression of E-cadherin, as well as increase of Vimentin, AEG-1 and p-GSK-3β, was clearly observed in tissues from metastatic lymph nodes. In the orthotopic lung cancer animal model, mice bearing Slu-01/AEG-1 cells also showed a significant increase in the number of lymph node metastases where cancer tissues clearly exhibited mesenchymal characteristics. These results strongly suggest that EMT should play a major role in the AEG-1-mediated metastasis of NSCLC.

Molecular mechanisms of EMT in lung cancer have been the most investigated fields. AEG-1 was found to control the expression level of Vimentin and E-cadherin. In some cancers, a mechanism involving the AEG-1-Vimentin interaction has been reported [16,31]. Recently, the role of Vimentin in EMT has also been reported in breast cancer cell lines, which was attributed to a mechanism involving the activation of AEG-1 [32]. In this study, we detected a typical EMT process induced by AEG-1 in NSCLC cells. However, EMT is regulated by various cell signaling pathways that originate from the tumor stroma, including TGF-β [33], Wnt [34], Hedgehog [35], Notch [36] and Ras-MAPK pathways [37]. Among these pathways, aberrant activation of Wnt/β-catenin signaling has been found in a wide range of cancers, especially in NSCLC [38]. Moreover, Wnt/β-catenin activation may induce EMT through its downstream targets: Twist, Snail and Slug. Previous studies have shown that Wnt/β-catenin signaling participates in EMT in numerous cancers; however, the phenotypes and downstream molecular events are fairly different, reflecting the dependence on cellular context and tissue specificity [39]. Our current data shows that AEG-1 can promote the accumulation of nuclear β-catenin. The activities of β-catenin luciferase reporter constructs were significantly decreased by AEG-1 siRNA in NCl-H226 cells. Furthermore, β-catenin could reverse AEG-1-siRNA’s effect on EMT. Consistent with the previous reports, we also found that both activation of β-catenin and promotion of EMT can result from up-regulaton of AEG-1, further supporting the notion that the β-catenin-mediated pro-EMT function of AEG-1 up-regulation might also contribute to AEG-1–induced metastasis in lung cancer. These data indicate that AEG-1 is a key promotor of EMT through activating Wnt/β-catenin signaling.

Wnt/β-catenin signaling pathway has been widely implicated as the regulator of cell invasion and migration in cancers [40,41]. β-catenin is a main downstream effector of the canonical Wnt signaling pathway, which has a dual role in EMT: it not only enhances cell-cell adhesion by associating with cadherin complexes in adherent junctions of cell membrane, but also functions as a transcriptional co-activator by interacting with TCF transcription factor complexes in the nucleus [42]. Yoo et al. reported that AEG-1 can activate the canonical Wnt signaling pathway [9]. Other studies demonstrated that many growth factors, such as insulin growth factor, transforming growth factor-β, and epidermal growth factor, could increase β-catenin accumulation through Ser-9 phosphorylation of GSK-3β [43]. Our analysis also revealed that Ser-9 phosphorylation of GSK-3β was involved in the stability and transcriptional activity of AEG-1-mediated β-catenin. Furthermore, the physical interaction between AEG-1 and GSK-3β facilitates GSK-3β inactivation through Ser-9 dephosphorylation, which increases nuclear β-catenin accumulation and transcriptional activity. These results revealed the potent promoting function of AEG-1 on Wnt/β-catenin signaling. However, since AEG-1 is not a phosphatase, the molecular mechanism of GSK-3βinactivation may be mediated by a separate phosphatase associated with this complex. Recent studies have also implicated CKI as a positive regulator of β-catenin signaling, which phosphorylates several components of the β-catenin degradation complex in vitro such as GSK-3β [44]. In our study, coimmunoprecipitation data indicated that AEG-1 could form a complex with GSK-3 and CKIδ. Thus, identification of small molecules that could perturb the interaction between AEG-1 and its partners, resulting the inhibition of AEG-1 function, might be a rational and effective way of target therapy of NSCLC.

In recent years, the prognostic value of AEG-1 has been widely confirmed in various cancers [45,46], and its tumor-promoting role has also been manifested. Our data provided evidence that high expression of AEG-1 was closely correlated with poor prognosis and lower patient survival rate. We concluded that the combined detection of the AEG-1 level and EMT status showed more significant prognostic value, suggesting that they may be regarded as correlative predictive factors for death in lung cancer patients. From the results of Kaplan-Meier analysis, we can conclude that AEG-1 is a reliable prognostic factor of the overall survival; moreover, AEG-1 combining with EMT status is able to more accurately predict the probability of death in lung cancer patients.

## Conclusions

In summary, this study first delineates the functional role of AEG-1 in EMT and metastasis of NSCLC, and demonstrates how AEG-1 underlies the onset of EMT and aggressive metastasis of lung cancer by activating Wnt/β-catenin signaling. These findings also uncover a novel molecular mechanism that maintains the constitutive activation of the Wnt/β-catenin signaling by AEG-1, and AEG-1 may prove to be clinically useful for developing a new prognostic biomarker and therapeutic target for lung cancer.
